# Upstream stimulatory factors are involved in the P1 promoter directed transcription of the AbetaH-J-J locus

**DOI:** 10.1186/1471-2199-9-110

**Published:** 2008-12-16

**Authors:** Alessia Finotti, Susan Treves, Francesco Zorzato, Roberto Gambari, Giordana Feriotto

**Affiliations:** 1 Department of Biochemistry and Molecular Biology, Molecular Biology Section, University of Ferrara, Via Fossato di Mortara 74, 44100 Ferrara, Italy; 2 Department of Experimental and Diagnostic Medicine, General Pathology Section, University of Ferrara, Via Borsari 46, 44100 Ferrara, Italy; 3 Departments of Anaesthesia and Research, Basel University Hospital, Hebelstrasse 20, 4031 Basel, Switzerland

## Abstract

**Background:**

Alternative splicing of the locus AβH-J-J generates functionally distinct proteins: the enzyme aspartyl (asparaginyl) β-hydroxylase (AAH), truncated homologs of AAH with a role in calcium homeostasis humbug and junctate and a structural protein of the sarcoplasmic reticulum membranes junctin. AAH and humbug are over expressed in a broad range of malignant neoplasms. We have previously reported that this locus contains two promoters, P1 and P2. While AAH and humbug are expressed in most tissues under the regulation of the P1 promoter, AAH, junctin and junctate are predominantly expressed in excitable tissues under the control of the P2 promoter. We previously demonstrated that Sp transcription factors positively regulate the P1 promoter.

**Results:**

In the present study, we extended the functional characterization of the P1 promoter of the AβH-J-J locus. We demonstrated by quantitative Real-time RT-PCR that mRNAs from the P1 promoter are actively transcribed in all the human cell lines analysed. To investigate the transcription mechanism we transiently transfected HeLa cells with sequentially deleted reporter constructs containing different regions of the -661/+81 P1 nucleotide sequence. Our results showed that (i) this promoter fragment is a powerful activator of the reporter gene in HeLa cell line, (ii) the region spanning 512 bp upstream of the transcription start site exhibits maximal level of transcriptional activity, (iii) progressive deletions from -512 gradually reduce reporter expression.

The region responsible for maximal transcription contains an E-box site; we characterized the molecular interactions between USF1/2 with this E-box element by electrophoretic mobility shift assay and supershift analysis. In addition, our USF1 and USF2 chromatin immunoprecipitation results demonstrate that these transcription factors bind the P1 promoter *in vivo*.

A functional role of USF1/USF2 in upregulating P1-directed transcription was demonstrated by analysis of the effects of (i) *in vitro *mutagenesis of the P1/E-box binding site, (ii) RNA interference targeting USF1 transcripts.

**Conclusion:**

Our results suggest that USF factors positively regulate the core of P1 promoter, and, together with our previously data, we can conclude that both Sp and USF DNA interaction and transcription activity are involved in the P1 promoter dependent expression of AAH and humbug.

## Background

We have previously characterized the human AβH-J-J locus, a genomic sequence which generates functionally distinct proteins [1], including the enzyme aspartyl (asparaginyl) β-hydroxylase (AAH), junctin, a structural protein of sarcoplasmic reticulum, humbug and junctate, the truncated homologs of AAH calcium binding proteins [1,2]. AAH catalyzes posttranslational hydroxylation of aspartate and asparagine residues in certain epidermal growth factor-like domains present in a number of proteins, such as receptors and receptor ligands, involved in cell growth and differentiation, and extracellular matrix molecules [3]. AAH, mediates cell motility and invasiveness, an effect which is of interest because of its role in placental implantation and "receptivity" of endometrium [4]. Humbug is a truncated homolog of AAH that lacks a catalytic domain. Overexpression of humbug increases intracellular calcium levels by promoting its release from intracellular stores [5]. The levels of humbug immunoreactivity are directly associated with colon cancer tumor grade and inversely associated with patient survival [6]. AAH and/or humbug are over expressed in infiltrative intrahepatic cholangiocarcinomas, metastasized lung, breast, colon, hepatocellular carcinomas, and malignant neuroectodermal tumors [7-11]. These proteins can contribute to the malignant phenotype by increasing motility and enhancing proliferation, survival, and cell cycle progression. Inhibition of AAH and humbug expression could represent an attractive approach for gene therapy of infiltrating tumors [3,11].

Junctate is an integral calcium binding protein of sarco(endo)plasmic reticulum membrane, which forms a supramolecular complex with the inositol 1,4,5 trisphosphate receptor and modulates calcium entry through receptor- and store-activated channels [1,12,13].

Our group previously reported the identification of two promoter sequences, present within the human AβH-J-J locus (named P1 and P2), that are expected to regulate the transcription of this locus [1,14,15]. The generated primary transcripts undergo alternative splicing and direct the synthesis of AAH, humbug, junctin, and junctate. We have previously reported the characterization of the P2 promoter, demonstrating that the myocyte enhancer factor 2 (MEF-2) transcription factor binds to this promoter sequence and drives tissue-specific expression, being responsible of inducing transcription during muscle differentiation [14,15].

In additon, we have recently reported the role of Sp factors in upregulating the P1-directed transcription of the AβH-J-J locus [16]. This was the first study about the role of the P1 promoter.

In the present study we focused our attention on the role of another putative regulating sequence, namely an E-box, which is located in the region of the P1 promoter and is required for high-level of transcription.

To characterize the expression directed by the P1 promoter, we have analysed the corresponding mRNAs in different cell lines. Furthermore, transfections of HeLa cells with progressively deleted reporter constructs of the -661/+81 P1 promoter region were performed and the transcription activity of each fragment was characterized. DNA/transcription factor interaction studies were performed *in vitro *by EMSA or supershift assays, and in intact cells by chromatin immunoprecipitations. Functional assays were performed by *in vitro *mutagenesis of the E-box binding site and by RNA interference targeting USF1 [17].

## Results

### Structure of the 5' end of the AβH-J-J locus

**Figure 1:**
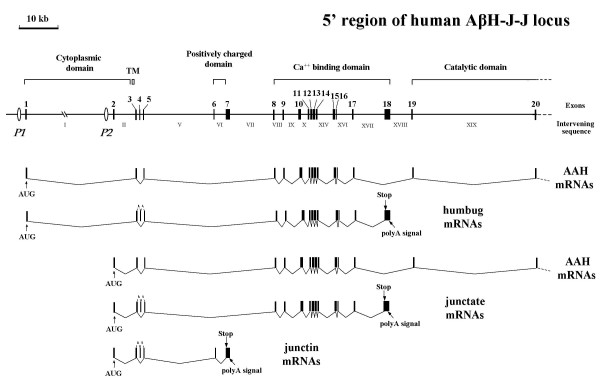
**Structure of the 5' end of the human locus for AAH, humbug, junctate, and junctin**. Arabic numbers over black boxes indicate exons. Intervening sequences are indicated in Roman numbers. The two putative promoters P1 and P2 are indicated. A schematic representation of AAH, humbug, junctate and junctin exons splicing is reported at the bottom of the panel. The cytoplasmic, TM, positively charged, calcium-binding, and catalytic domains are indicated. The locations of AUG, stop codons, and poly(A) signals are shown.

**Figure 2:**
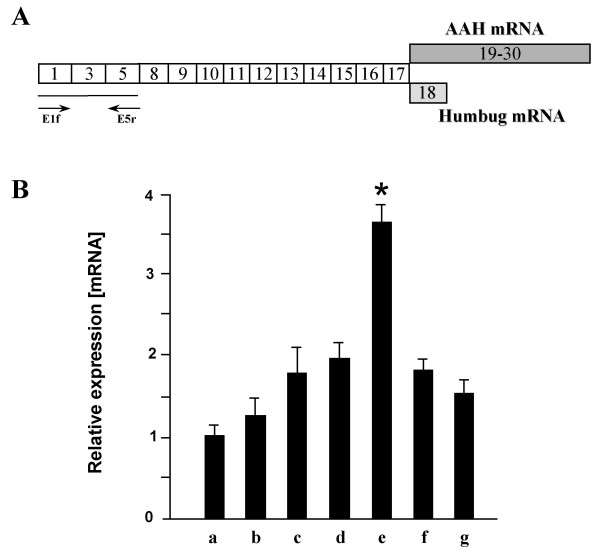
**Quantitative Real-time RT-PCR analysis of the transcripts starting from P1 promoter in human cell lines**. (A) The exon 1 starting mRNAs of AAH and humbug, the PCR primers and the 297 bp PCR product are represented; white boxes indicate exons common to the two transcripts. (B) Relative mRNA expression levels of P1 promoter transcripts were evaluated by Quantitative real-time RT-PCR in which cDNA derived from human cell lines (a, MCF7; b, Colo 38; c, Hek293; d, HepG2; e, HeLa; f, RD; g, MDA) total RNA were amplified. Relative quantification is expressed as normalized fold expression and was calculated by using MCF7 as reference sample (value = 1). All data represent the mean of three determinations from two independent experiments; *P < 0.001.

Figure 1 shows the structure of the 5' end of the human AβH-J-J locus and the transcripts for AAH, humbug, junctate and junctin, with the location of the P1 and P2 promoters. Figure 2 shows the quantitative Real-time RT-PCR analysis of the transcripts starting from P1 promoter in a panel of human cell lines. In this experiment cDNA derived from MCF7, Colo 38, Hek293, HepG2, HeLa, RD and MDA human cell lines were employed. The results obtained indicate that the P1 promoter directs transcription in all the cell lines analysed and is more active in HeLa cells (Figure 2, column e). These data confirm and further extend the concept that P1 promoter, unlike the tissue-specific P2 promoter, directs transcription in cells of different tissues, as recently reported by our research group [15,16].

### Identification of functional region within the AβH-J-J P1 promoter

In order to identify putative regulatory regions located within AβH-J-J P1 promoter, HeLa cells were transiently transfected with sequentially deleted reporter constructs, containing different regions of the -661/+81 P1 nucleotide sequence cloned into pGL3-basic reporter vector [16].

**Figure 3:**
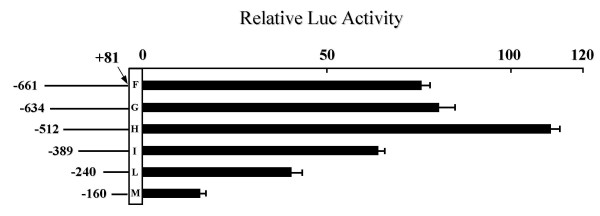
**AβH-J-J P1 promoter activity in HeLa cell line**. HeLa cells were transiently transfected with sequentially deleted reporter constructs of the -661/+81 P1 nucleotide sequence (represented in the left side of the figure, F-M). Transient transfection and luciferase assays were performed in triplicate, and the data (right side of the figure) were normalized to renilla luciferase activity and are shown as relative activities compared to that for pGL3-basic, a reporter vector with a basal promoter. The values are the means ± SD of at least three independent experiments.

**Figure 4:**
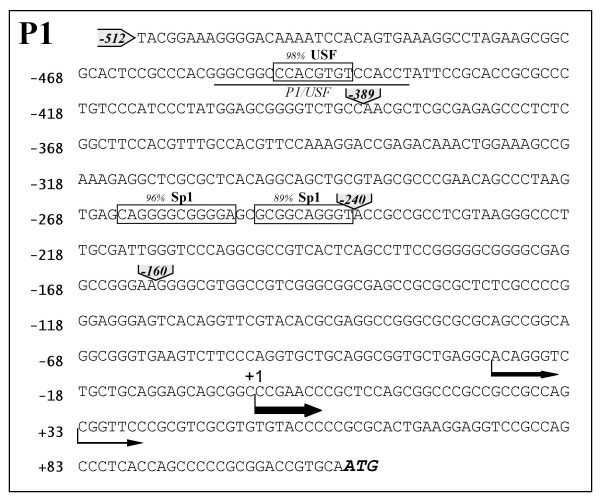
**DNA sequence of the AβH-J-J P1 promoter region**. (A) -512/+112 P1 promoter and 5' UTR sequences. Solid line indicate the oligonucleotide used in EMSA (Table 1). Sequence homologies to USF and Sp1 transcription factor binding site are boxed; the percent of homology was obtained by *TF SEARCH *ver. 1.3. Arrows indicate the characterized transcription initiation sites (arrow thickness is proportional to transcription start activity) [16]. The nucleotide deletions of reporter constructs are shown in grey.

The data of transient transfection and luciferase assays were normalized to renilla luciferase activity. An additional comparison was done with the pGL3-basic reporter vector. The results represented in Fig 3 demonstrate that: i) the -661/+81 promoter region exhibits high reporter gene expression in HeLa cells; ii) luciferase activity is higher with the reporter construct lacking the -634/-512 sequence, suggesting the presence in this region of cis-acting elements inhibiting transcription; iii) the expression of the reporter gradually decreases when the sequence from -512 to -160 is progressively removed. These results clearly demonstrate that the maximal luciferase activity is dependent on the -512/+81 sequence (Figure 3H construct), indicating the presence within this region of positive regulatory elements, responsible for the maximum level of transcription activity. In order to identify putative binding sites for transcription factors, computer-assisted analysis of the sequences was performed. The -512/+1 region displays sequence homology to the following transcription factor binding sites: USF1 (98%, Fig 4), AP-1 (88%), GATA-1/3 (90–85%), NF-E2 (91%) and Sp1 (98–81%). We have previously reported the interaction of Sp transcription factors to P1 promoter [16]; we therefore analyzed the other putative binding sites by EMSA, demonstrating that only the USF1 element is able to bind nuclear extracts.

### *In vitro *and *in vivo *Interaction of USF1 and USF2 transcription factors with the AβH-J-J P1 promoter

**Figure 5:**
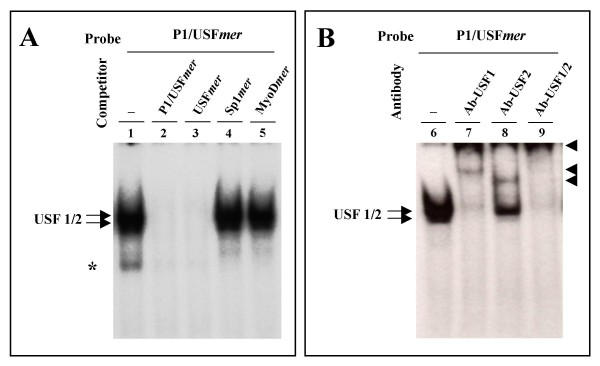
**Interaction of USF1 and USF2 transcription factors with the AβH-J-J P1 promoter**. (A) EMSA were carried out on 2 μg of Hela nuclear extracts as described in the Experimental procedures using P1/USF*mer *probe (Table 1). Probe was incubated with nuclear extracts in the absence of competing oligonucleotides (lane 1) or in the presence of the indicated competing oligonucleotides (lanes 2–5). Arrows and asterisk indicate the specific and non-specific complexes respectively. (B) Supershift assay was performed using P1/USF*mer *probe and 2 μg of nuclear extract from HeLa cells; probe was incubated with nuclear exctract in the absence of antibody (lane 6) or in the presence of antibodies against USF1 factor, USF2 factor or both (lanes 7, 8, and 9 respectively). Arrows indicate the specific complexes and arrowheads indicate the super-shifted complexes.

To determine whether the E-box site is able to interact with USF1 and USF2 transcription factors, EMSA experiments were carried out using 2 μg of HeLa nuclear extracts and the P1/USF*mer *probe (Table 1). The results obtained are reported in Figure 5 and indicate that the P1/USF*mer *probe stably interacts with nuclear extracts, forming the complexes shown in figure 5A (lane 1, arrows). As expected, P1/USF*mer *and consensus USF*mer *probes efficiently compete with the binding (Figure 5A, lanes 2, 3), while Sp1*mer *and MyoD*mer *probes do not (Figure 5A, lanes 4, 5). In order to demonstrate the presence of USF1 and USF2 transcription factors in the retarded complexes, supershift analyses were performed. We incubated P1/USF*mer *probe and 2 μg of nuclear extract from HeLa cells with antibodies against USF1, USF2 or both factors (Figure 5B, lanes 7, 8 and 9 respectively). Our results indicate that both antibodies induce a supershift of the retarded bands. These data conclusively demonstrate that both USF1 and USF2 are able to bind to the P1/USF*mer *probe in vitro.

**Table 1:** Sequence of synthetic oligonucleotides used in this study

Double-stranded oligonucleotides used in gel shift assays and mutagenesys^a^		
*P1/USFmer*		5'-GGCGGCC***CACGTG***TCCACCT-3'
*USFmer*^b^		5'-GGCCAGAC***CACGTG***GTCTGTTC-3'
*Sp1mer*^b^		5'-CCCTTGGTGGGGGCGGGGCCTAAGCTGCG-3'
*MyoDmer*^c^		5'-CCCCCCAACACCTGCTGCCTGA-3'
*P1/USFmut*		5'-CCCACGGGCGGCC*ACA*GTGTCCACCTATTC-3'
*P1/Spmut*		5'-GTGAGCA*TA*G*T*CG*TA*GAGC*AT*GG*T*AG*A*GTACCGC-3'

Q-PCR Primers	Amplified region	Sequences

*P1 ChIP f*	P1 promoter AβH-J-J	5'-AACGAACTAAATTCCTGCTTCAGC-3'
*P1 ChIP r*	(contains USF elements)	5'-TCCTTTGGAACGTGGCAAACGT-3'
		
*Neg ChIP f*	negative control region	5'-TGTGTGATTTCCCGTCAACTGTC-3'
*Neg ChIP r*	(without USF elements)	5'-CCAGCCTCTTCCATTGGATACAA-3'
		
*E1 f*	from exon 1 to 5	5'-CAAGAGCAGCGGCAACAG-3'
*E*5*r*	of AβH-J-J transcripts	5'-AATAAAACTTTGGCATCATCCACTCAAAATCTCC-3'
		
*qUSF1 f*	USF1 transcripts	5'-TGATGATGCAGTTGACACGGA-3'
*qUSF1 r*		5'-CAGTTGTTGATCTTGTCTCGGC-3'
		
*GAPDH f*	glyceraldehyde-3-phosphate	5'-AAGGTCGGAGTCAACGGATTT-3'
*GAPDH r*	dehydrogenase mRNA	5'-ACTGTGGTCATGAGTCCTTCCA-3'
		
*HMBS f*	hydroxymethyl-bilane	5'-GAACATGCCCTGGAGAAGAATGA-3'
*HMBS r*	synthase mRNA	5'-GGTAGCCTGCATGGTCTCTTGTAT-3'
		
*ACTIN f*	actin mRNA	5'-TGACGGGGTCAACCACACTGTGCCCATCTA-3'
*ACTIN r*		5'-CTAGAAGCATTTGCGGTGGACGATGGAGGG-3'

^a ^Nucleotide mutations are underlined; ^b^The oligonucleotide containing USF1 and Sp1sites were purchased from Geneka; ^c ^[14].

**Figure 6:**
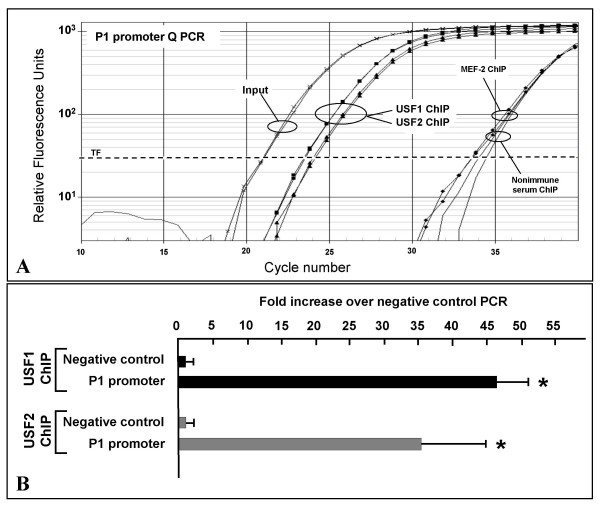
**Chromatin immunoprecipitation assay performed to evaluate in vivo USF interaction with P1 promoter**. (A) Representative quantitative real-time PCR profiles for the amplification of the P1 promoter in the ChIP assay. *USF1 ChIP*, *USF2 ChIP *and *MEF-2A ChIP *represents duplicate amplification curves from chromatin immunoprecipitated with USF1, USF2 or MEF-2A antiserum respectively; *nonimmune serum *represent curves from immunoprecipitations with nonimmune rabbit serum. *Input *represents curves obtained from HeLa chromatin (1%) before immunoprecipitation. TF, threshold fluorescence. (B) *In vivo *association of USF1 and USF2 transcription factors with the AβH-J-J P1 promoter. The results, obtained from ChIP assay quantitative real time PCR using USF1 (USF1 ChIP) or USF2 (USF2 ChIP) antiserum, were analyzed following the methodology described in the *methods *section. The fold increase over negative control PCR in each case compares the values obtained by P1 promoter amplification to the corresponding amplification of a distal genomic region lacking USF1 binding sites (Table 1). All data represent the mean of two separated PCR experiments each performed in triplicate, and obtained from at least two independent immunoprecipitation. The asterisk indicates that the value is significantly different (p < 0,05) from the control value.

In order to verify whether USF1/2 and P1 promoter interact in vivo, chromatin immunoprecipitation (ChIP) assays were performed on HeLa cells. Chromatin was immunoprecipitated using either USF1 and USF2 antiserum and quantitative amplification of P1 promoter was performed on purified DNA. A representative ChIP assay (Figure 6A) shows that amplification curves from samples immunoprecipitated with USF1 or USF2 antisera reach threshold 10 cycles early than those treated with MEF-2 antiserum or non immune serum. In figure 6B ChIP results are reported as immunospecific fold enrichment in P1 promoter sequences relative to a negative control sequence that lacks USF1 binding sites. The data supports the in vivo interaction between USF1 and USF2 and the P1 promoter in intact cells.

### Mutation of the USF element of the AβH-J-J P1 promoter: effects on transcription

**Figure 7:**
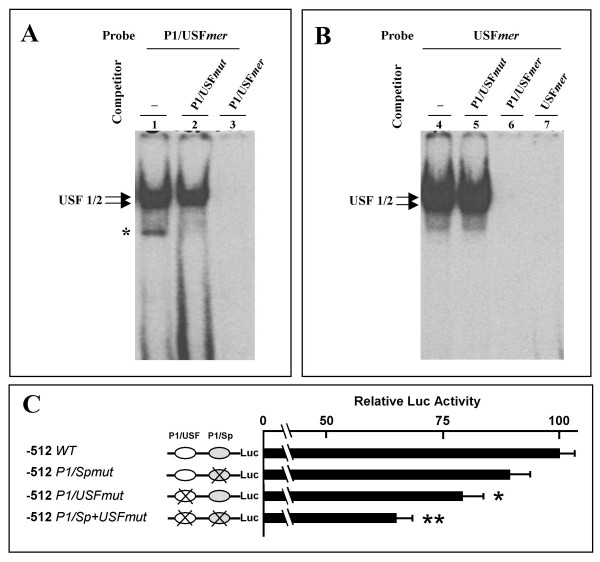
**Mutational analysis of USF elements in the AβH-J-J P1 promoter**. (A) EMSA were performed incubating P1/USF*mer *probe with 2 μg of nuclear extract from HeLa cells in the absence (lane 1) or in the presence of 100 fold molar excess of the indicated competing oligonucleotides (lanes 2–3). (B) USF*mer *probe was incubated with 2 μg of nuclear extract from HeLa cells in the absence (lane 4) or in the presence of 100 fold molar excess of the indicated competing oligonucleotides (lanes 5–7) (Table 1). Arrows and asterisks indicate the specific and aspecific complexes respectively. (C) The reporter construct -512 WT (H in Fig 3) was subjected to site directed mutagenesis in the sequence spanning Sp1 and USF binding sites. The constructs obtained were designated as -512 *P1/Spmut *and -512 *P1/USFmut*. In addition a double mutant containing both mutations was indicated as -512 *P1/Sp+USFmut*. HeLa cells transient transfection and luciferase assay were performed in two experiments conducted in triplicate and the data were normalized to *Renilla *luciferase activity and reported as ratios (means ± SD) to the wild type reporter construct -512 WT; *P < 0.05 and **P < 0.02.

Figure 7A–B shows the effect of the mutation of the P1/USF*mer *probe on its ability to bind nuclear factors. ^32^P-labelled P1/USF*mer *(Figure 7A) or USF*mer *(Figure 7B) probes were incubated with HeLa cell nuclear extract in the absence or in the presence of 100 fold molar excess of competing wild type or mutant (P1/USF*mut*) oligonucleotides (see Table 1 for nucleotide sequences). The data obtained demonstrate that the mutations fully suppress the ability of the probes to bind nuclear factors (Figure 7A, lane 2 and Figure 7B, lane 5). The effect of this mutation on the transcription activity are shown in Figure 7C. The reporter construct -512/+81 (H in Fig 3) was subjected to site directed mutagenesis in the sequence spanning Sp1 and USF1 binding sites (constructs -512 *P1/Spmut *and -512 *P1/USFmut*). In addition a double mutant containing both mutations was generated (-512 *P1/Sp+USFmut*). HeLa cells were transfected with wild type -512/+81 (-512 WT), single or double mutant AβH-J-J P1 promoter reporter constructs. Comparison of reporter expression in HeLa extracts shows that the mutation in the USF binding site significantly inhibits transcription directed by the -512 construct (Figure 7C). This effect is further enhanced when the -512 *P1/Sp+USFmut *double mutant construct is employed. These data strongly suggest that interaction of nuclear factors to the USF binding site is required for maximum level of P1 promoter transcription.

### Effect on P1 promoter activity of USF1 silencing obtained by RNA interference

**Figure 8:**
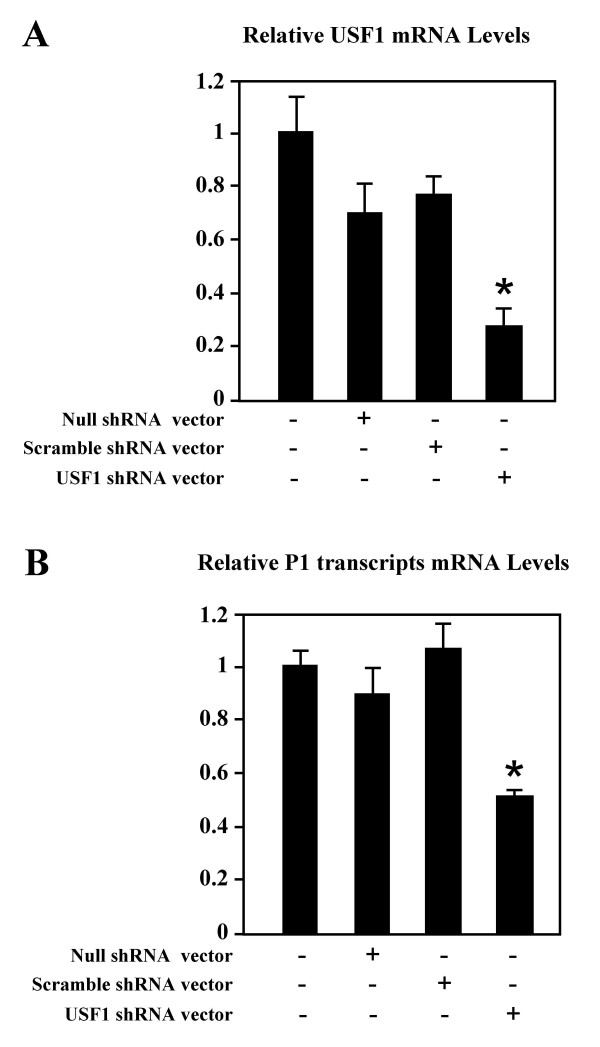
**Effect of USF1 silencing obtained by short interference RNA on P1 promoter activity**. HeLa cells were transiently transfected with USF1 shRNA vector, with Scramble shRNA vector or vector in which no small hairpin DNA oligo was cloned (Null shRNA vector). Two days after transient transfection of these vectors into HeLa cells, total RNA was extracted and used for quantitative Real Time RT-PCR, using primers targeting USF1 mRNA (panel A) or P1 promoter specific transcripts (panel B). Results represent the average ± SD of three independent experiments carried out in duplicate; ΔΔCt method was used to compare gene expression data, standard error of the mean was calculated; statistical significance: *P < 0.05.

In order to demonstrate the role of proteins belonging to the USF family on the transcription directed by the P1 promoter of the human AβH-J-J locus, silencing of the USF1 gene was performed using short interfering RNAs. A double stranded oligonucleotide, targeting human USF1 RNA sequence, or a scrambled sequence (Table 2), which has no significant homology to human genes and transcripts, were cloned into pSingle-tTS-shRNA plasmid. HeLa cells were then transiently transfected with USF1 shRNA vector. To detect non-specific effects, scramble shRNA vector and construct lacking small hairpin DNA (Null shRNA) were used as controls. Two days after transfection, total RNA was extracted and used for quantitative Real Time RT-PCR, using primers targeting USF1 mRNA or P1 promoter specific transcripts. Figure 8A shows that USF1 mRNA levels are strongly reduced following transfection with USF1 shRNA vector. In addition Figure 8B shows that USF1 depletion was associated with inhibition of the P1 directed transcription. The effects of null shRNA and scramble shRNA vectors were much lower or absent on USF1 mRNA levels (Figure 8A) and P1 directed transcription (Figure 8B) respectively.

**Table 2:** Double stranded oligonuleotides sequences inserted in shRNA vectors

**USF1 shRNA**
5'-*TCGAG*GTCGACAACAGGTGGAAGATTTCAAGAGAATCTTCCACCTGTTGTCGACTTTTTTACGCGT*A*-3'

3'-*C*CAGCTGTTGTCCACCTTCTAAAGTTCTCTTAGAAGGTGGACAACAGCTGAAAAAATGCGCA*TTCGA*-5'

**Scramble shRNA**

5'-*TCGAG*GTACCGAACAACGCAACGAATTCAAGAGATTCGTTGCGTTGTTCGGTACTTTTTTACGCGT*A*-3'

3'-*C*CATGGCTTGTTGCGTTGCTTAAGTTCTCTAAGCAACGCAACAAGCCATGAAAAAATGCGCA*TTCGA*-5'

The 19 base pair oligonucleotides specific for USF1 siRNA or containing scambled sequences are underlined; XhoI and HindIII cloning sites are in italic.

## Discussion

The aim of the present paper is to investigate in detail one of the two promoter sequences regulating the transcription of the AβH-J-J locus [14,15]. We isolated and identified the 5'-flanking region of the exon 1 of this locus. The cloned nucleotide sequence allowed us to characterize the P1 promoter region, which is involved in the regulation of AAH and humbug expression. We have been able to identify transcripts relative to P1 promoter activity in all tissues and cell lines analyzed [15,16]. Similar to many housekeeping gene promoters, the region under investigation lacks a TATA box and an initiator element [18]. In contrast, this sequence is GC-rich and presents homologies with Sp1 consensus binding site, which we previously demonstrated to play a role in transcription [16]. Transfection experiments with progressively deleted P1 promoter reporter constructs in HeLa cells showed that maximal promoter activity is located within 512 nucleotides from the principal transcription initiation site (see Figures 3), a result which confirms our previous data in HepG2 and RD cell lines [16]. Computer-assisted analysis of the -512/+1 region indicates the presence of sequence homologies to binding sites for USF1, Sp1 and for other transcription factors. EMSA analyses demonstrated no binding activity for these identified elements with the exception of USF1 and Sp1 sites [16]. In the present study we analyzed the DNA binding activity and function of USF transcription factors on P1 promoter.

The first conclusion of this manuscript derives from EMSA assays, supershift analysis and chromatin immunoprecipitation assays, which coherently demonstrated that USF1 and USF2 factors bind to the P1/USF site of the P1 promoter, both in vitro (Figures 5) and in intact cells (Figure 6).

The second conclusion is the demonstration that these interactions are functionally relevant for transcription efficiency. The relevance of the USF binding for the transcription regulation of the P1 promoter has been addressed by two complementary approaches: (a) mutagenesis of the P1/USF element and (b) use of a USF1 silencing approach.

The results obtained concurrently demonstrate that USF transcription factors and USF1 binding site are involved in the up-regulation of the P1 promoter of the human AβH-J-J gene locus. On the other hand, mutagenesis experiments point out that the residual transcription activity of the double mutant is compatible with the presence of other transcription factors participating in the regulation of the P1 promoter. This is in agreement with previously published results from our group [16] demonstrating the presence of at least 12 Sp binding sites and Sp1/3 dependent transcription activity of P1 promoter. In addition, it is in agreement with the experiments on progressively deleted reporter constructs indicating that the decrease of transcription associated with the -512/-389 deletion is higher (Figure 3, – 42%) than that of the single mutant -512 *P1/USFmut *construct with respect to the wild type plasmid (Figure 7, – 21%). This evidence coherently sustain the concept that other regulatory factors, in addition to USF, are involved in P1 promoter directed transcription of the AβH-J-J locus.

When the results presented here are taken together with those recently published by Feriotto et al. [16], it appears that both Sp and USF DNA interactions and transcription activities are involved in the regulation of this human locus [19-23].

Comparison of the P1 and P2 promoter sequences of the AβH-J-J locus reveals important differences are clearly detectable [15]. The most interesting result emerging from studies focused on the P2 promoter is that the calcium-dependent transcriptional factor MEF-2 activates the transcription of junctin, junctate and AβH in striated muscles and brain [14]. Neither Sp1 nor USF binding sites were detected within the P2 promoter. On the other hand, P1 promoter, which drives the expression of AAH and humbug in most tissues, contains one USF1 and several Sp1 functionally active binding sites within the sequence having maximal transcription activity [15,16]. The finding that the sequences present in the upstream P1 promoter are significantly different from those of the P2 promoter is, in our opinion, of great interest.

From the practical point of view, the impact of USF proteins in the transcriptional regulation of this locus will be of future interest considering the potential contribution of AAH and humbug to the infiltrating growth of neoplasms by increasing cell migration and enhancing proliferation and survival [5-11]. For instance, overexpression of humbug and AAH is associated with malignant progression in human gastric cancer cells [24]. In addition, relevant to our study, Xian et al. found that the protein expression level of AAH in the hepatocellular carcinoma was parallel with the mRNA expression level, indicating that the expression is regulated at the transcription level [25]. Inhibition of AAH and humbug transcription could represent an attractive approach for gene therapy of infiltrating tumors [3,5]. Of course, in discussing the possible implications of targeting mRNAs for USF transcription factor(s), careful considerations are required, due to a wide involvement of this transcription factor in the regulation of expression of several genes, including genes involved in development and cell cycle progression [17].

## Conclusion

When the results shown in the present paper are considered together with previously published reports by our research group, it can be concluded that the AβH-J-J locus contains at least two functionally distinct promoters (P1 and P2), and multiple alternative splicing sites, leading to the synthesis of the functionally distinct proteins, AAH, humbug, junctin, and junctate [1,14-16]. The P1 promoter directs AAH and humbug expression in most tissues and is up-regulated by Sp and USF transcription factors.

## Methods

### Cell Cultures

The human hepatoblastoma, HepG2, breast cancer, MCF7 and MDA-MB231, cervix epithelial carcinoma, HeLa, and embryonic kidney, Hek293, were cultured in E-MEM (Sigma, St. Louis, MO, USA) supplemented with 2 mM L-glutamine (BioWhittaker, Walkersville, MD, USA) and 1 mM sodium pyruvate (Sigma); embryonic rhabdomyosarcoma, RD cell line was cultured in D-MEM ((BioWhittaker) supplemented with 4 mM L-glutamine; melanoma, Colo 38, cell line was cultured in RPMI 1640 (Sigma). All cell lines were grown in a humidified atmosphere containing 5% CO_2 _at 37°C in medium supplemented with 100 units/ml penicillin, 100 μg/ml streptomycin, and 10% (vol/vol) FBS.

Cell lines were all obtained from the ATCC (American Type Culture Collection, Manassas, VA, USA).

### RT-PCR Real-time

Total RNA was harvested from cell lines by using the TRIzol^® ^Reagent (Invitrogen, Carlsbad, CA, USA). cDNA was synthesized from 0.5 μg of total RNA using ImProm-II™ (Promega, Madison, WI, USA).

For quantitative real-time PCR reaction, 0,5/20 μl aliquots of cDNA, 10 pmol of *E1f *and *E5r *primer (Table 1), 1 × iQ™ SYBR^® ^Green Supermix (Bio-Rad, Hercules, CA, USA), were used for each Sybr Green real-time PCR reaction to quantitate the relative expression of P1 specific transcripts. Primer *E1f *and *E5r *were designed to amplify a 297 bp sequence present in all the transcripts relative to P1 promoter activity and spanned intronic sequences in order to rule out amplification from genomic DNA. Primers pairs and amplification conditions were validated by melting curve and electhrophoretic analysis. Real-time PCR reactions were performed for a total of 40 cycles (95°C for 10 s, 65°C for 30 s, and 72°C for 50 s) using an iCycler IQ^® ^(Bio-Rad). The relative proportions of each template amplified were determined by using IQ5 software (Bio-Rad), employing the ΔΔCt method to compare gene expression data. Each sample was quantitated in triplicate from at least two independent experiments. Mean ± S.D. values were determined for each fold difference. Amplification of human Actin, GAPDH and HMBS cDNA has been used as internal standards (housekeeping genes). Negative controls (no template cDNA) were also run with every experimental plate to assess specificity and to rule out contamination.

### Transient transfection and dual luciferase assay

HeLa cells were transiently transfected with sequentially deleted reporter constructs, obtained as previously described [16], containing different regions of the -661/+81 P1 nucleotide sequence cloned into pGL3-basic reporter vector upstream firefly luciferase.

Cells were seeded at 70–80% confluence in 16-mm wells. Transfection was performed after 24 hours using 1.2 μg of Lipofectamine™ 2000 (Invitrogen), 30 ng pRL-TK vector (Promega), which contains the *Renilla *luciferase gene as a transfection efficiency control and 0.6 μg of *Firefly *luciferase reporter plasmid per well. Lysates were prepared 48 h after transfection by adding 100 μl of passive lysis buffer (Dual Luciferase^® ^Reporter Assay System, Promega). Luciferase activity was determined with an analytical luminometer (model TD-20/20, Turner Designs, Sunnyvale, CA, USA); the light intensity produced by *Firefly *luciferase (test plasmid) was normalized to that produced by *Renilla *luciferase (control plasmid). Promoter activity was expressed as *fold induction *relative to that of cells transfected with pGL3-basic vector [16].

### Nuclear extract preparation

Nuclear extracts were prepared as described by Andrews and Faller [26]. Briefly, cells were collected, washed twice with ice-cold phosphate-buffered saline, and resuspended in 0.4 ml/10^7 ^cells of hypotonic lysis buffer (10 mM Hepes/KOH, pH 7.9, 10 mM KCl, 1.5 mM MgCl_2_, 0.5 mM dithiothreitol, and 0.2 mM phenylmethanesulfonyl fluoride). After incubation on ice for 10 min, the mixture were vortexed for 10 seconds, and nuclei were pelleted by centrifugation at 12000 *g *for 10 seconds, then nuclear proteins were extracted by incubation of the nuclei for 20 min on ice with intermittent gentle vortexing in 20 mM Hepes/KOH, pH 7.9, 25% glycerol, 420 mM NaCl, 1.5 mM MgCl_2_, 0.2 mM EDTA, 0.5 mM dithiothreitol, 0.2 mM phenylmethanesulfonyl fluoride, 1 μg·mL^-1 ^aprotinin, 1 μg·mL^-1 ^leupeptin, 2 mM Na_3_VO_4_, and 10 mM NaF (Sigma, St Louis, MO, USA); cell debris was removed by centrifugation at 12000 *g *for 5 min at 4°C. The Bradford method was used to measure the protein concentration in the extract, which was then stored in aliquots at -80°C.

### Electrophoretic mobility shift assays

The double-stranded oligonucleotides (ODN) used in the EMSA are reported in Table 1. 3 pmol of ODN were ^32^P-labeled using OptiKinase (GE Healthcare, Chalfont St Giles, UK), annealed to an excess of complementary ODN and purified from [γ-^32^P]ATP (Perkin Elmer, Wellesley, MA, USA). Binding reactions were performed by incubating 2 μg of nuclear extract and 16 fmol of ^32^P-labeled double-stranded ODN, with or without competitor in a final volume of 20 μL of binding buffer (20 mM Tris-HCl, pH 7.5, 50 mM KCl, 1 mM MgCl_2_, 0.2 mM EDTA, 5% glycerol, 1 mM dithiothreitol, 0.01% TritonX100, 0.05 μg·μL^-1 ^of poly dI-dC, 0.05 μg·μL^-1 ^of a single-stranded ODN) [14]. Competitor (100 fold excess of unlabeled ODNs) and nuclear extract mixture were incubated for 15 min and then probe was added to the reaction. After a further incubation of 30 min at room temperature samples were immediately loaded onto a 6% nondenaturing polyacrylamide gel containing 0.25 × Tris/borate/EDTA (22.5 mM Tris, 22.5 mM boric acid, 0.5 mM EDTA, pH 8) buffer. Electrophoresis was carried out at 200 V. Gels were vacuum heat-dried and subjected to autoradiography. Supershift assays were performed as described previously [16] by using 2 μg of commercially available antibodies specific for USF1 (sc-229X) and USF2 (sc-862X) transcription factors (Santa Cruz Biotechnology Inc., Santa Cruz, CA, USA).

### Chromatin Immunoprecipitation Assays

Chromatin immunoprecipitation assays were performed by using the Chromatin Immunoprecipitation Assay Kit (Upstate Biotechnology Inc., Lake Placid, NY, USA). Briefly, a total of 6 × 10^7 ^HeLa cells (from 5 15-cm plates) were treated, for 10 min at room temperature, with 1% formaldehyde culture medium. Cells were washed in phosphate-buffered saline, and then glycine was added to a final concentration of 0.125 M. The cells were then suspended in 1.5 ml of lysis buffer (1% SDS, 10 mM EDTA, and 50 mM Tris-Cl, pH 8.1) plus protease inhibitors (1 μg/ml pepstatin A, 1 μg/ml leupeptin, 1 μg/ml aprotinin, and 1 mM phenylmethylsulfonyl fluoride) and the chromatin subjected to sonication (using a Sonics Vibracell VC130 sonicator with a 2-mm probe). Fifteen 15-s sonication pulses at 30% amplitude were required to shear chromatin to 200–1000 bp fragments. 0.2-ml aliquots of chromatin were diluted to 2 ml in ChIP dilution buffer containing protease inhibitors and then cleared with 75 μl of salmon sperm DNA/protein A-agarose 50% gel slurry (Upstate Biotechnology) for 1 h at 4°C before incubation on a rocking platform with either 6–10 μg of specific antiserum USF1, USF2, and MEF-2A (Santa Cruz Biotechnology) or normal rabbit serum (Upstate Biotechnology). 20 μl of diluted chromatin was saved and stored for later PCR analysis as 1% of the input extract). Incubations occurred overnight at 4°C and continued an additional 1 h after the addition of 60 μl protein A-agarose slurry. Thereafter the agarose pellets were washed consecutively with low salt, high salt and LiCl buffers. DNA/protein complexes were recovered from the pellets with elution buffer (0.1 M NaHCO3 with 1% SDS), and cross-links were reversed by incubating overnight at 65°C with 0.2 M NaCl. The samples were treated with RNase A and proteinase K, extracted with phenol/chloroform and ethanol-precipitated. The pelletted DNAs were washed with 70% ethanol and dissolved in 40 μl of Tris/EDTA. 2 μl aliquots were used for each real-time PCR reaction to quantitate immunoprecipitated promoter fragments.

### Real-time PCR Quantitation of Immunoprecipitated Promoter Fragments

For quantitative real-time PCR reaction conditions each 25 μl reaction contained 2 μl of template DNA (from chromatin immunoprecipitations), 10 pmol of primers (Table 1) and 1 × iQ™ SYBR^® ^Green Supermix (Bio-Rad). Real-time PCR reactions were performed for a total of 40 cycles (97°C for 15 s, 65°C for 30 s, and 72°C for 30 s) using an iCycler IQ^® ^(Bio-Rad). The relative proportions of immunoprecipitated promoter fragments were determined based on the threshold cycle (*Tc*) value for each PCR reaction. Real time PCR data analysis followed the methodology previously described [27,28]. A Δ*TC *value was calculated for each sample by subtracting the *Tc *value for the input (to account for differences in amplification efficiencies and DNA quantities before immunoprecipitation) from the *Tc *value obtained for the immunoprecipitated sample. A ΔΔ*Tc *value was then calculated by subtracting the Δ*Tc *value for the sample immunoprecipitated with specific antiserum from the Δ*Tc *value for the corresponding control sample immunoprecipitated with nonimmune rabbit serum. Fold differences (specific antiserum ChIP relative to nonimmune serum control ChIP) were then determined by raising 2 to the ΔΔ*TC *power. PCR was also performed using control primers that amplify a 197 bp genomic region lacking USF1 binding sites. The ΔΔ*Tc *values were then calculated as previously described and the negative control PCR data were used to normalize quantitative results from different immunoprecipitation. Each sample was quantitated in triplicate on at least two separate experiments and from at least two independent immunoprecipitations. Mean ± S.D. values were determined for each fold difference.

### Site-directed mutagenesis

Mutagenesis was performed by using a QuickChange site-directed mutagenesis kit (Stratagene, La Jolla, CA, USA). Two double stranded mutant oligonucleotides (*P1/Spmut *and *P1/USFmut *plus the flanking 9–12 mer of P1 nucleotide sequence (Table 1) were used to inactivate specific binding in the pGL3 construct containing the -512/+81 promoter sequence. The nucleotide sequence of the mutant constructs was confirmed by DNA sequencing.

### USF1-targeted shRNA plasmid construction and transfection in HeLa cells

The effect of USF1 on P1 promoter directed expression was evaluated by RNA interference approach using the Knockout™ Single Vector Inducible RNAi System (Clontech, Mountain View, CA, USA). siRNA targeting USF1 mRNA (GenBank accession n° NM_007122) were designed by using the *siRNA-sequence designer *software (Clontech). We produced a double strand oligonucleotide for shRNA plasmid construction containing from 5' to 3' end: XhoI cloning site, a guanine residue, the 19 base pair target specific sense strand followed by a short spacer (TTCAAGAGA), the reverse complement of the sense strand, six thymidines, as an RNA polymerase III transcription stop signal, a HindIII cloning site (Table 2). This oligonucleotide was cloned into XhoI-HindIII site of the pSingle-tTS-shRNA Vector (Clontech), in which shRNA is expressed under the control of the U6 promoter. A negative control scrambled siRNA, which had no significant homology to human gene and transcript sequences, was designed to detect any non specific effects.

After cloning and sequence characterization, 500 ng of plasmids were transfected into HeLa cells using Lipofectamine™ 2000 (Invitrogen), and, after 8 hours, 20 ng/ml of doxycycline were added. Fourty-eight hours later RNA extraction and reverse transcription were performed. To quantitate the depletion of USF1 and AβH-J-J P1 directed transcripts, 1/20 μl aliquots of cDNA and 10 pmol of the primers reported in table 1 were used for each Sybr Green real-time PCR reaction. Resuls were normalized with glyceraldehyde-3-phosphate dehydrogenase (GAPDH) and hydroxymethylbilane synthase (HMBS) expression levels (housekeeping genes). Real-time PCR reactions were performed for a total of 40 cycles (95°C for 10 s, 66°C for 30 s, and 72°C for 50 s). The ΔΔCt method was used to compare gene expression data.

### Statistical analysis

All the data were normally distributed and presented as mean ± S.D. Statistical differences between groups were compared using one-way ANOVA (ANalyses Of VAriance between groups) software. Statistical significance was assumed at p < 0.05.

## List of abbreviations

AAH: aspartyl (asparaginyl) β-hydroxylase; PCR(s): polymerase chain reaction(s); bp: base pair(s); MEF-2: myocyte enhancer factor 2; USF: Upstream Stimulatory Factor; Sp: specific transcription factor; EMSA: electrophoretic mobility shift assay; ChIP: chromatin immunoprecipitation; siRNA: short interference RNA; shRNA: short hairpin RNA.

## Authors' contributions

AF planned and performed all the experiments and participated in the design and writing of the manuscript. ST collaborated in mutant reporter vectors design, production, and validation. FZ participated in the P1 reporter constructs production and contributed to the transfection experiments. RG participated in the analysis of the results and critically reviewed the manuscript. GF designed the study, supervised the experiments and wrote the manuscript. All authors read and approved the final manuscript.
